# Peptides with Dual Antimicrobial and Anticancer Activities

**DOI:** 10.3389/fchem.2017.00005

**Published:** 2017-02-21

**Authors:** Mário R. Felício, Osmar N. Silva, Sônia Gonçalves, Nuno C. Santos, Octávio L. Franco

**Affiliations:** ^1^Instituto de Medicina Molecular, Faculdade de Medicina, Universidade de LisboaLisbon, Portugal; ^2^S-Inova Biotech, Pós-graduação em Biotecnologia, Universidade Católica Dom BoscoCampo Grande, Brazil; ^3^Programa de Pós-Graduação em Patologia Molecular, Universidade de BrasíliaBrasília, Brazil

**Keywords:** anticancer peptides (ACPs), antimicrobial peptides (AMPs), cancer, multi-resistant infections, bacteria

## Abstract

In recent years, the number of people suffering from cancer and multi-resistant infections has increased, such that both diseases are already seen as current and future major causes of death. Moreover, chronic infections are one of the main causes of cancer, due to the instability in the immune system that allows cancer cells to proliferate. Likewise, the physical debility associated with cancer or with anticancer therapy itself often paves the way for opportunistic infections. It is urgent to develop new therapeutic methods, with higher efficiency and lower side effects. Antimicrobial peptides (AMPs) are found in the innate immune system of a wide range of organisms. Identified as the most promising alternative to conventional molecules used nowadays against infections, some of them have been shown to have dual activity, both as antimicrobial and anticancer peptides (ACPs). Highly cationic and amphipathic, they have demonstrated efficacy against both conditions, with the number of nature-driven or synthetically designed peptides increasing year by year. With similar properties, AMPs that can also act as ACPs are viewed as future chemotherapeutic drugs, with the advantage of low propensity to resistance, which started this paradigm in the pharmaceutical market. These peptides have already been described as molecules presenting killing mechanisms at the membrane level, but also acting toward intracellular targets, which increases their success compartively to one-target specific drugs. This review will approach the desirable characteristics of small peptides that demonstrated dual activity against microbial infections and cancer, as well as the peptides engaged in clinical trials.

## Introduction

At the beginning of the twenty-first century, the increased appearances of multi-resistant bacterial pathogens have become a worldwide problem (Arias and Murray, 2009). The World Health Organization has already emphasized the urgency in designing new antimicrobial molecules, because conventional antibiotics are increasingly useless as therapeutics, especially against the so-called *ESKAPE* pathogens (*Enterococcus faecium, Staphylococcus aureus, Klebsiella pneumoniae, Acinetobacter baumanii, Pseudomonas aeruginosa*, and *Enterobacter* species), which showed a high propensity to develop antibiotic resistance (McKenna, 2013). Another global concern is the rise in the incidence of cancer. Recent data released revealed 12.7 million new cases and 7.6 million deaths, just in 2008 (Ferlay et al., 2010). In Europe alone, 3.45 million new cases were diagnosed and 1.75 million deaths occurred during 2012 (Ferlay et al., 2013). Nowadays, cancer is the second most common cause of death worldwide (Arnold et al., 2015), caused by an abnormal cellular growth, in a uncontrolled manner, with the ability to invade other tissues, leading to the formation of tumor masses, neo-vascularization (angiogenesis), and metastasis (Thundimadathil, 2012). Lung, colorectal, prostate, and breast cancer are the most diagnosed forms of this disease (Domalaon et al., 2016). Considering the numbers revealed, it is urgent to find new anticancer drugs able to control tumor growth with minimal side effects (Dennison et al., 2007). This situation has become worse due to DNA-alkylation, hormone agonists, and antimetabolites, which show insufficient selectivity and unspecific targeting on healthy cells (Smith and White, 1995; Gaspar et al., 2013), contributing to increased resistance to anticancer drugs (Wang K.-r. et al., 2009). Moreover, the intersection between infection and cancer is highlighted by the number of cancer deaths and new occurrences that are related to treatment or chronic infections. Approximately 2 million of the new cancer patients are due to infectious agents like bacteria and viruses (Parkin, 2006; Vedham et al., 2014; Attiê, 2014). Patients that suffer from a chronic infection are more susceptible to cancer due to the weakened immune system, which cannot fight both the pathogen, and the emergence of cancer cells (Rolston, 2001). This weakness can also occur due to cancer treatments that are too aggressive to patient health, such as chemotherapy, radiotherapy, and surgical resection, leaving patients susceptible to infection agents (Fishman, 2011; Xiao et al., 2015). Also, continuous exposure to infection leads to inflammation, contributing to the appearance of cancer (Vedham et al., 2014^2014^).

In recent years, a promising new class of molecules has arisen, and it has different types of advantages against both of the above major world health concerns. Antimicrobial peptides (AMPs) are small peptides essential for the innate immune response of organisms of all branches, presenting activity against a wide range of pathogens, like bacteria, fungi, and viruses (Hancock et al., 2016). More recently, anticancer activity was also described for some of these peptides, termed anticancer peptides (ACPs) (Dennison et al., 2006). Properties like their short time-frame of interaction (which decreases the probability of resistance), low toxicity (which reduces side effects), mode of action, specificity, good solubility, and finally, good tumor penetration, indicate ACPs as a future chemotherapy cancer drug with high potential (Riedl et al., 2011; Figueiredo et al., 2014; Wu et al., 2014; Gaspar et al., 2015; Domalaon et al., 2016).

## Peptides with antimicrobial and anticancer activity

Antimicrobial peptides were first identified due to their importance in the innate immunity of a broad number of organisms, gaining interest from the scientific community (Jenssen et al., 2006). From the first identification until today, hundreds of AMPs have been identified and studied, either from natural sources or from *in silico* designs (Hancock et al., 2016). These peptides are characterized by an amino acid sequence usually from 5 to 50 residues, high hydrophobicity and positive net charge (Melo et al., 2011; Gaspar et al., 2012). These physicochemical properties set the basis for the activity against pathogens (Dennison et al., 2010). Bacteria present negatively charged membranes, promoting AMPs' initial electrostatic interaction. Even knowing that not all AMPs are ACPs, the similarity in terms of action is obvious, due to the phenotype of the membrane surface in cancer cells. In the plasma membrane inner-leaflet of healthy cells there is phosphatidylserine (PS), a negatively charged phospholipid. This asymmetry between inner and outer membrane leaflets is lost in cancer cells, leading to the presence of PS in the outer-leaflet (Bevers et al., 1996). PS exposure, the presence of O-glycosylated mucins, sialylated gangliosides, and heparin sulfate, in conjugation with an increased transmembrane potential, surface area, and membrane fluidity (Schweizer, 2009; Hilchie et al., 2011), promote the specific activity of AMPs toward cancer cells (ACPs), without being affected by tumors' heterogeneity (Kelly et al., 2016).

The physicochemical parameters determining the activity of some AMPs toward cancer cells are still unclear, considering that the characteristics of AMPs/ACPs are very similar. Efforts are being made in order to understand these differences, which would enable an improved design of ACPs (Dennison et al., 2006). Some AMPs can also be ACPs independently of the source of identification or synthetic route of design (Mader and Hoskin, 2006). The number of AMPs encountered in nature that have anticancer activity has increased in recent years. Aurein 1.2 (GLFDIIKKIAESF), a peptide isolated from the frog *Litoria aurea*, is one example of an AMP with broad-range activity toward bacteria that showed to be highly active toward 55 different cancer cell lines *in vitro*, without any significant cytotoxic activity (Rozek et al., 2000; Dennison et al., 2007; Giacometti et al., 2007). Another example is the human neutrophil peptide-1 (HNP-1, ACYCRIPACIAGERRYGTCIYQGALWAFCC), an AMP that plays a fundamental role in the defense against pathogens in the innate immune system. Its antimicrobial activity has been fully explored, with a broad spectrum activity against bacteria, but it is the possibility of using this AMP in cancer therapies that attracted attention in recent years (Nishimura et al., 2004; Varkey and Nagaraj, 2005). The full mechanism of action of this peptide against cancer cells has not yet been established, but activity was already confirmed for different cancer cell lines, with very low cytotoxicity against healthy cells (McKeown et al., 2006; Gaspar et al., 2015). Peptides pleuricidin 03 (GRRKRKWLRRIGKGVKIIGGAALDHL) and pleuricidin 07 (RWGKWFKKATHVGKHVGKAALTAYL), AMPs isolated from Atlantic flatfishes, were showed to be highly effective in killing different bacterial strains (Patrzykat et al., 2003). Recently, their anticancer activity was explored and their effectiveness against drug-resistant breast cancer cells confirmed, without toxicity against fibroblasts or erythrocytes, either in *in vitro* and *in vivo* models (Hilchie et al., 2011). These are just examples of ACPs that were studied after isolation from different natural sources, like animals, plants, and bacteria. Natural ACPs, even having a high anticancer activity, have normally 30–40 amino acids in their sequence, which increases production costs. Therefore, synthetic routes for ACP design have gained attention. There are different possible approaches available, such as the improvement of natural ACP sequences or the use of *in silico* methods (Park et al., 1998; Lee et al., 2008). Both strategies take into consideration the improvement of the physicochemical properties, like amphipathicity, hydrophobicity, and overall positive charge, with the objective of better activity toward the target cells (Huang et al., 2011; Melo et al., 2011; Sinthuvanich et al., 2012). Furthermore, other strategies such as hybridizing different ACPs (Hoskin and Ramamoorthy, 2008) or changing the amino acids used for unnatural ones (D-enantiomers or cyclic tetra-substitution of C^α^ are examples; Hicks, 2016) have also been tested. The possibilities are endless, and depend on what the focus of the improvement is for each case. Bioinformatic algorithms integrated with machine learning, where the design is automatic through the properties chosen, taking into consideration AMP/ACP libraries of existing molecules, are considered the future method for their rational design (Tyagi et al., 2013; Lin et al., 2015).

**Figure 1:**
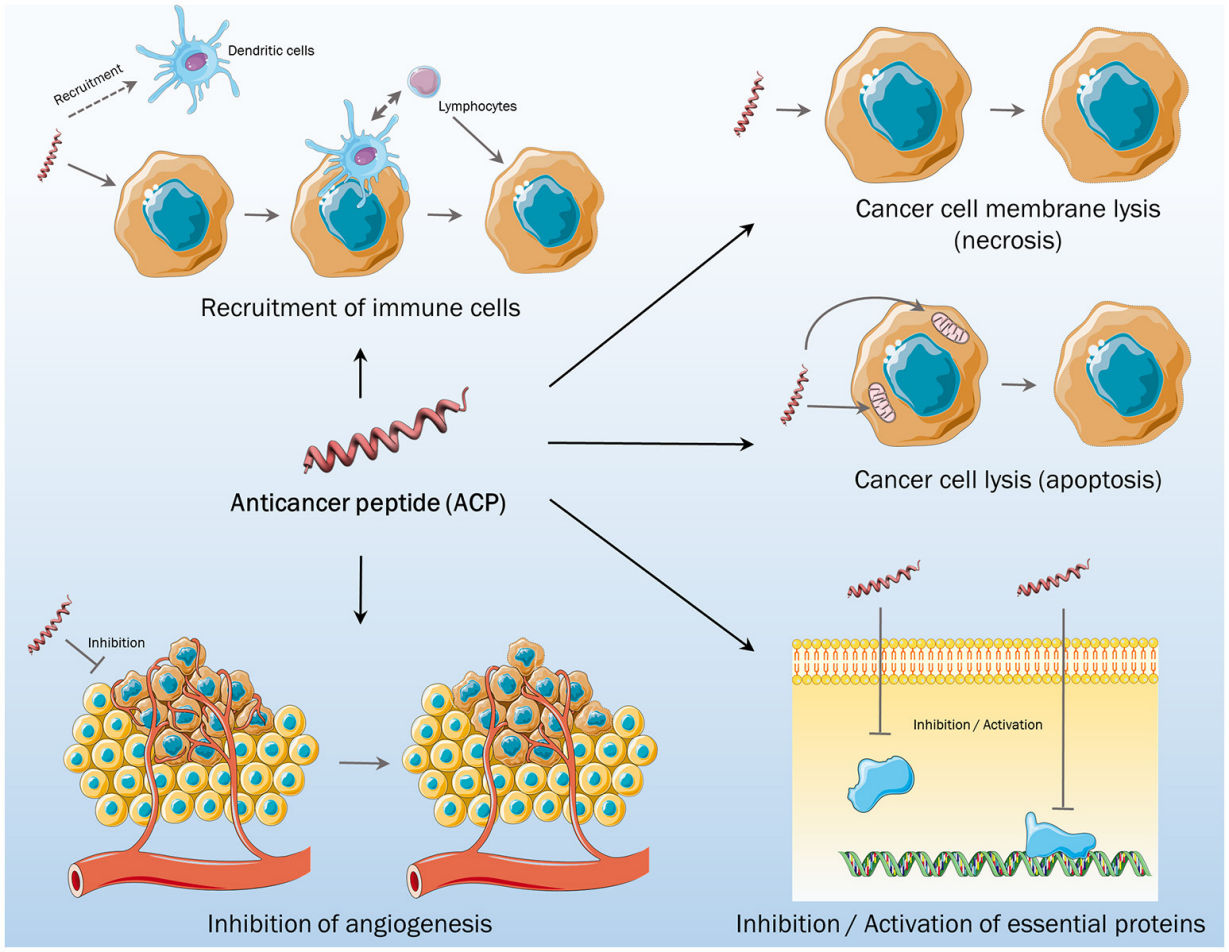
**Different mechanisms of action of anticancer peptides**.

AMPs and ACPs share most of the characteristics, like the physicochemical properties already described. Structure plays a central role in their activity. It is commonly accepted that most AMPs/ACPs do not fold in a well-defined structure when free in solution, but adopt α-helix or β-sheet structure when electrostatic interactions with membranes occur (Hoskin and Ramamoorthy, 2008). Differences in terms of structure were the first method for the classification of ACPs. Examples of some AMPs lately defined as α-ACPs are cecropin, magainin, melittin, and buforin II, with lactoferricin B, HNP-1/3, and gamesin being classified as β-ACPs (Papo and Shai, 2005). More recently, it was noticed that independently of the secondary structure that the peptide adopts, a classification considering the mechanisms of action in the target cancer cells was more suitable (Wu et al., 2014). AMPs were considered membrane-active peptides regarding their primary activity, but over the years, it was clarified that they can also target different processes of the pathogen (namely, metabolism, and cell division) and of the immune system (recruitment of immune cells; Hancock et al., 2016). These aspects were also studied for ACPs, with the identification of cell membrane lytic activity (necrosis), mitochondrial membrane lytic activity (apoptosis), and non-membrane activities (Figure 1; Wu et al., 2014). The first one is the most common anticancer method of targeting, with the electrostatic interactions promoting membrane disruption, leading to necrosis. Polybia-MPI, a natural ACP, and the synthetic BTM-P1 are just two examples (Segura et al., 2007; Wang K.-r. et al., 2009). These ACPs have high selectivity toward cancer cell membranes and develop low resistance, when compared to conventional chemotherapeutic drugs. Activity toward mitochondrial membrane, activating apoptosis signaling, was also observed for some ACPs, such as lactoferricin B and different β-ACPs (Furlong et al., 2006; Paredes-Gamero et al., 2012). After the activity at the membrane level, ACPs can also present other activities, either targeting essential cell proteins, inhibiting angiogenesis, or recruiting immune cells to attack cancer cells (Figure 1; Wu et al., 2014). HNP-1 was shown to be an ACP that recruits and activates dendritic cells in terms of immunomodulatory activity (Wang Y.-s. et al., 2009), but also inhibits angiogenesis, which is essential to the growth and development of tumors (Xu et al., 2008).

## Potential clinical approaches using ACPs

Although a wide variety of drugs are commercially available, treatments for infections, and cancer have one thing in common: the emergence of resistance against multiple drugs (Baguley, 2010; Theuretzbacher, 2012). Another associated problem is the lack of selectivity of the available drugs, and their consequent undesirable side effects for the patients (Mandell et al., 2001; Baguley, 2010). Thus, there is a need for the development of new antineoplastic and antimicrobial therapies, with higher selectivity, leading to fewer side effects than current ones. It is desirable that these new compounds present different mechanisms of action, without dependence on activity toward a single specific molecule in the target cells, like the ones used nowadays in therapeutics. The main goal is resistance prevention, overcoming the existing mechanisms that cancer and bacterial cells use, being active and diminishing the side effects (Lincke et al., 1990; Arias and Murray, 2009; Kakde et al., 2011).

As described earlier, several AMPs and/or ACPs have become the focus of research by different groups, mainly due to their ability to kill or inhibit the growth of a variety of microorganisms and tumor cells (Wu et al., 2014; Hancock et al., 2016). There are thousands of natural peptides and millions of synthetic peptides obtained by rational design, with a large number presenting antimicrobial and anticancer activity, but only a few being tested (Gordon et al., 2005). Furthermore, from these, unfortunately, only a small number are currently in clinical trials (Table 1). This is mostly due to the numerous challenges associated with the development of these peptides as pharmaceutical drugs, such as synthesis costs, which are higher than the synthesis of organic antibiotic small molecules. Due to this, peptide design has focused on primary structure shortening, accomplishing a lower production cost, and allowing physicochemical properties to be easily changed, which is important for the activity of AMPs/ACPs (Tørfoss et al., 2012a; Domalaon et al., 2016).

**Table 1:** **Anticancer and antimicrobial peptides in clinical trials, with the indication of the highest phase and the therapeutic condition for which they are being tested**.

**Product name**	**Peptide**	**Company**	**Highest phase**	**Condition treated**	**Route of administration**
**ACPs**					
ANG-4043	ANG-4043	AngioChem Co.	Preclinical	Brain metastases	IV
CLS-001	MBI-226	Cadence Pharmaceuticals Inc.	II	Vulvar intraepithelial neoplasia	IV
		Carrus Capital Corp.			
		Cutanea Life Sciences Inc.			
		Migenix Inc.			
GRN-1201	GRN-1201	Green Peptide Co.	I	Solid tumors	IV
ICT01-2588	ICT01-2588	Incanthera Ltd.	I	Vascular disrupting agents	IV
		University of Bradford		Breast cancer (preclinical)	
				Colorectal cancer (preclinical)	
				Lung cancer (preclinical)	
				Prostate cancer (preclinical)	
ICT03-Es5	ICT03-Es5	Incanthera Ltd.	I	Solid tumors	IV
		University of Salford		Breast cancer (preclinical)	
				Liver cancer (preclinical)	
				Non-small cell lung cancer (preclinical)	
ICT04-CYP	ICT04-CYP	Incanthera Ltd.	Preclinical	Bladder cancer	IV
		University of Bradford		Colorectal cancer	
ITK-1	ITK-1	FUJIFILM Co.	III	Glioblastoma	IV
		Green Peptide Co.		Prostate cancer	
		Kurume University			
Oncopore™	LTX-315	Lytix Biopharma AS	I	Solid tumors	IV
Paclitaxeltrevatide	ANG-1005	AngioChem Co.	II	Brain metastases	IV
				Glioblastoma	
				Glioma	
WT-2725	WT-2725	Sumitomo Dainippon Pharma Co.	I	Hematological malignancies	IV
		Sunovion Pharmaceuticals Inc.		Solid tumors	
**AMPs**					
C16G2	C16G2	Chengdu Sen Nuo Wei Biotechnology Co.	II	Dental caries	Topical
		C3 Jian Inc			
Cefilavancin®	TD-1792	GlaxoSmithKline Co.	III	Gram-positive infections	Topical
		Theravance Biopharma Inc.		Skin and soft tissue infections	
		R-Pharm			
CLS-001	MBI-226	Cadence Pharmaceuticals Inc.	III	Rosacea	Topical
		Carrus Capital Corp.		*Acne vulgaris* (II)	
		Cutanea Life Sciences Inc.		Genital warts (II)	
		Migenix Inc.			
Dalvance™	MDL-63,397	Durata Therapeutics Inc.	II	Osteomyelitis	IV
		Pfizer Inc.		Osteomyelitis (I)	
		Vicuron Pharmaceuticals Inc.		Pneumonia (preclinical)	
DPK-060	DPK-060	DermaGen AB	II	Atopic dermatitis	Topical
		Pergamum AB		Otitis externa	
		Karolinska Development AB			
LL-37	LL-37	Pergamum AB	II	Leg ulcer	Topical
		Karolinska Development AB			
Locilex®	MSI-78	Dipexium Pharmaceuticals Inc.	III	Diabetic foot ulcer	Topical
		Genaera Corp.		Skin and soft tissue infections (I)	
		GlaxoSmithKline Plc.			
		RRD International Inc.			
Luminaderm®	NP108	NovaBiotics Ltd.	II	Bovine mastitis	Topical
Lytixar^TM^	LTX109	Lytix Biopharma AS	II	Impetigo	Topical
				*Staphylococcus aureus* infections	
Murepavadin®	POL-7080	Polyphor Ltd.	II	*Pseudomonas aeruginosa* infections	IV
		University of Zurich		Gram-negative infections (I)	
Novamycin®	NP-339	NovaBiotics Ltd.	I	Cystic fibrosis	IV
				Invasive fungal disease	
				Oropharyngeal candidiasis	
Novarifyn®	NP-432	NovaBiotics Ltd.	Preclinical	Methicillin-resistant *Staphylococcus* aureus (MRSA) *P. aeruginosa*	IV
				*C. difficile* infections	
Novexatin^®^	NP-213	NovaBiotics Ltd.	II	Onychomycosis	Topical
		Taro Pharmaceutical Industries Ltd.			
NVB302	NVB302	Novacta Biosystems Ltd.	I	*C. difficile* infections	Topical
PXL-01	Lactoferrin	DermaGen AB	III	Post-surgical adhesions	Topical
		Karolinska Development AB			
		PharmaSurgics AB			
		Promore Pharma			
		Pergamum AB			
SGX-942	Dusquetide	Inimex Pharmaceuticals Inc.	Preclinical	Melioidosis	IV
		SciClone Pharmaceuticals Inc.			
		Soligenix Inc.			
		University of British Columbia			
Surotomycin	MK-4261	Cubist Pharmaceuticals Inc.	III	*Clostridium difficile* infections	IV
		Merck & Co. Inc.			
Telavancin^®^	TD-6424	Clinigen Group plc	III	Osteomyelitis	IV
		Innoviva Inc.		Bacterial infections (I)	
		Pendopharm			
		Theravance Biopharma Inc.			
		University of Illinois			

*The search was carried out in the investigational drug databases Pharmaprojects (www.pharmaprojects.com), AdisInsight (www.adisinsight.com), Prous DDR (http://www.prous.com), and IDdb3 (http://science.thomsonreuters.com)*.

In addition, the adverse effects presented by some peptides (high toxicity to mammalian healthy cells and low immune response modification) increase the number of obstacles to applying these molecules to therapy (Hancock, 1997; Andreu and Rivas, 1998; Xiao et al., 2015; Kao et al., 2016). This is not surprising, since the activity of AMPs/ACPs usually depends on membrane-peptide interaction. However, to be commercially useful, it would be necessary to dissociate the toxicity to the mammalian cells from antimicrobial/antitumor activity, which can be achieved by increasing antimicrobial activity, reducing haemolytic activity, or both (Chen et al., 2005; Uggerhøj et al., 2015).

Another obstacle to the applicability of peptides is their susceptibility to proteolysis. Oral administration remains the preferred mode for drug delivery, corresponding to approximately 60% of the administration routes used for drugs (Renukuntla et al., 2013). This occurs due to the advantages that these drugs present, including low production cost and patient compliance in the administration. Even so, peptide drugs usually follow the traditional route of administration, like intramuscular (i.m.) or intravenous (i.v.) injection, due to their poor oral bioavailability, which is expressed by a low resistance to proteases and poor penetration through the intestinal membrane (Hamman et al., 2005). Sensitivity to proteolytic degradation can be mitigated by using rational design to replace naturally occurring amino acids with unnatural ones (Gordon et al., 2005; Uggerhøj et al., 2015). An example is the synthetic design of D-enantiomeric peptides, like DJK-5/6, which show improved activity against bacterial infections in *in vivo* models, comparable to that of the L-enantiomeric peptides, without showing any cytotoxic activity (de la Fuente-Núñez et al., 2015; Mansour et al., 2016). This type of peptide were also shown to be more actively effective against drug-resistant tuberculosis pathogens, and have already been tested with inhalable spray-dried formulations (Lan et al., 2014; Kwok et al., 2015). In terms of ACPs, SVS-1 was seen to be more effective, compared to its L-isomeric peptide form (Sinthuvanich et al., 2012). β^2, 2^ amino acids, also unnatural ones, can be another strategy to design AMPs/ACPs that are resistant to proteolysis, with a high effectiveness against the target cells and low toxicity toward healthy cells (Tørfoss et al., 2012a,b).

Together with proteolysis comes the limitation of pharmacokinetics and pharmacodynamics, because it is difficult to evaluate the direct action of the peptide against the pathogen *in vivo* and relate to a specific mode of action (Drusano, 2004). Moreover, the time of circulation, which is essential for a drug to be efficient, is not easy to determine (Kelly et al., 2016). Different strategies have been proposed for this problem, like the use of drug carriers, such as bacteriophages (Dąbrowska et al., 2014). Using a natural bacterial phage, displaying ACPs on their surface, increases the targeting (dynamics of action) and allows for improved dual activity. Conjugating the peptide with cell-penetrating peptides (CPPs) can be another interesting strategy to improve the specificity of the targeting. Some authors have used TAT protein from HIV virus as the CPP, conjugated to an AMP/ACP (HPRP-A1) in order to increase the specificity toward cancer cells (Hao et al., 2015). Coating or conjugation of peptides with polymers, like polyethylene glycol (PEG), can also increase circulation and improve pharmacokinetics/dynamics, independently of the polymer used, by allowing a higher time of circulation and improving their penetration toward the target cancer cells (Kelly et al., 2016).

In conclusion, these modifications may promote changes in amphipathic/hydrophobic properties, leading to the reduced cytotoxicity of peptides toward mammalian cells, without jeopardizing antimicrobial/anticancer efficiency, rendering peptides more impervious to proteolysis, and thus bestowing on them improved therapeutic activity and pharmaceutic design (Chen et al., 2005; Uggerhøj et al., 2015; Kang et al., 2017).

## Conclusion and future directions

In conclusion, AMPs and ACPs have been known for several decades, but only in the last one an increasing number of publications on thier *in vivo* activities has arisen. Consequently, few peptides are used in medical practice. However, we believe that in the upcoming years peptides will have a major impact on the treatment of infectious diseases and cancer, two of the world's greatest healthcare concerns. As shown here, different microbial infections and/or cancer-targeting peptides are in clinical trials, with approval for clinical application expected for the next few years (at least 10 in the next 5 years). Moreover, that number should tend to increase due to advances in the rational design of peptides, minimizing or eliminating cytotoxic effects. In addition, advances in the large-scale synthesis of peptides has made this process cheaper, thus making peptide-based therapies likely to become more accessible to patients. Another strategy that has gained attention is the combined use of peptides with conventional drugs, which reduces costs per treatment, minimizing the problem of resistance and preventing recurrence. Thus, AMPs and ACPs have great potential, both alone and in combination with conventional drugs, to be used in infection and cancer therapies, mostly due to their effective mechanisms of action on the target cells.

## Author contributions

MF, OS, SG, and NS wrote the article. SG, NS, and OF reviewed the article.

### Conflict of interest statement

The authors declare that the research was conducted in the absence of any commercial or financial relationships that could be construed as a potential conflict of interest.
